# Toward Ethical Governance of Artificial Intelligence (AI)-Enabled Cognitive Monitoring in Aging Populations

**DOI:** 10.7759/cureus.101618

**Published:** 2026-01-15

**Authors:** Hana Abbasian

**Affiliations:** 1 Center for Bioethics, Harvard Medical School, Boston, USA

**Keywords:** aging brain, artifical intelligence, cognitive assessment, digital health awareness, ethics in ai, older adult

## Abstract

AI-enabled cognitive monitoring is increasingly being integrated into geriatric care, enabling continuous assessment of behavioral and cognitive patterns that can detect early cognitive changes. This editorial examines key ethical and governance challenges associated with these tools, including the epistemic opacity of machine-learning models, distributed clinical responsibility, dynamic consent for passive data collection, and the equitable performance of algorithms across diverse populations. It argues that addressing these challenges requires governance frameworks that clarify accountability, ensure interpretability, and protect patient autonomy while supporting clinical decision-making. By discussing these considerations, this piece provides a structured perspective on responsible innovation in AI-supported cognitive monitoring, advancing discourse on ethical integration of emerging digital tools in aging populations.

## Editorial

AI-enabled cognitive monitoring is emerging as a key application of machine learning in geriatric care, particularly as clinicians increasingly adopt continuous, multimodal assessments that analyze longitudinal behavioral and cognitive data to detect changes in cognitive function among older adults [1]. These AI systems use longitudinal phenotyping, which involves the continuous collection and analysis of behavioral and cognitive data, such as speech patterns, fine motor movements, daily activity rhythms, and interactions with digital devices, across extended periods to detect subtle changes in cognition and function that may indicate early stages of cognitive decline [1]. As these tools are more frequently implemented in healthcare, clinicians and researchers should establish data governance structures that ensure these technologies are used safely and equitably in clinical practice [2].

AI-based systems similarly rely on longitudinal phenotyping, defined as the continuous characterization of cognitive and behavioral traits over time, which can create significant clinical value but also necessitates new ethical and data governance guidelines [3]. These innovations require regulatory frameworks that clearly distinguish between diagnostic aids used during clinical visits and continuous monitoring tools that may operate passively in the background [2,3]. Each category raises distinct expectations for clinician response, patient consent, and algorithmic oversight. Another governance challenge is the epistemic opacity of machine learning models, meaning that the internal logic and decision-making processes of these algorithms are often not readily observable or easily understandable to clinicians and patients [2].

This lack of transparency can complicate clinical accountability and decision-making, but it may be mitigated through strategies such as the use of model interpretability tools, standardized validation protocols, and the transparent reporting of algorithmic outputs. Recent advances in AI design are improving model interpretability, including tools that provide insights into how specific inputs influence outputs and frameworks for presenting algorithmic decisions in ways that clinicians can readily understand [3]. Despite these ongoing efforts and technical progress, translating such interpretability into routine clinical practice remains a challenge, particularly in complex, longitudinal monitoring scenarios [2,3]. Moreover, continuous cognitive monitoring algorithms can detect subtle biomarkers and behavioral patterns that are not directly observable by clinicians [3,4], which further complicates clinical accountability and decision-making.

An important governance priority is the implementation of standards for algorithms’ ability to perform reliably across diverse populations and environments [2,3]. Cognitive monitoring models may be trained on datasets that do not adequately reflect linguistic, cultural, and educational variation, which can influence the extracted speech or behavioral features [3]. Regulators need to require performance testing across subgroups defined by age, language, mobility level, or comorbidities to ensure that performance remains consistent across diverse older adult populations.

AI-generated alerts derived from speech or mobility patterns should be accompanied by structured workflows that guide clinician responses. Without such guidelines, AI systems risk creating clinical ambiguity by failing to clarify how outputs should be integrated into medical decision-making. Institutions developing governance models may need to clearly define when AI-generated notifications of cognitive change should trigger a follow-up visit, neuropsychological testing, or additional monitoring, so that these digital tools effectively support clinical decision-making.

Additionally, institutions using cognitive monitoring tools must address the challenge of distributed clinical responsibility [2,5]. Distributed clinical responsibility refers to the allocation of obligations across multiple actors in the healthcare system when AI systems generate continuous or high-volume outputs [2]. This includes legal responsibility, meaning who is ultimately liable for clinical decisions; ethical accountability, meaning who is morally obligated to act in the patient’s best interest; and workflow-level responsibility, meaning who is expected to monitor, interpret, or escalate AI-generated alerts in day-to-day clinical operations. When AI systems produce high-frequency alerts or identify behavioral anomalies, it becomes unclear who is obligated to interpret, escalate, or document them [4,5]. Therefore, regulations must establish transparent responsibility frameworks that specify who is responsible for monitoring the system and validating algorithmic outputs.

Another crucial dimension involves the development of consent frameworks applicable to passive data collection through microphones, accelerometers, or home-based devices [2,3]. Because many cognitive monitoring tools function continuously, governance structures should incorporate dynamic and ongoing consent processes to reflect the evolving nature of autonomy in aging populations [3]. This may include periodic consent reaffirmation, clear explanations of what data are being monitored, and options for user control over data types and usage.

Finally, because some cognitive monitoring models rely on systems that adjust their parameters as new data are acquired, governance must address how such adaptive tools are systematically supervised and evaluated over time [2,5]. This could involve clearly defined requirements for monitoring subtle changes in algorithm behavior as input patterns evolve, so that systems remain stable and aligned with clinical standards even as they learn from new data.

AI-supported cognitive monitoring holds substantial promise for detecting early cognitive changes, supporting personalized care plans, and assisting clinicians as populations age. However, this editorial is conceptual in nature and does not present original empirical data, jurisdiction-specific regulatory analyses, or direct input from clinicians, patients, or other stakeholders. As such, the normative recommendations offered here should be understood as guidance for future research and discussion. Ultimately, realizing this potential will depend on establishing governance structures that protect patient autonomy and maintain clinical integrity. Through thoughtful regulation, these tools can become reliable collaborators in the long-term care of older adults, advancing cognitive health with greater precision and ethical clarity.
